# U1 Small Nuclear Ribonucleoprotein Autoantibodies Reflect the Disruption of the Blood–Nerve Barrier in Guillain–Barré Syndrome

**DOI:** 10.3390/ijms27146117

**Published:** 2026-07-08

**Authors:** Fumitaka Shimizu, Michiaki Koga, Nanami Yamanaka, Masayuki Nakamori

**Affiliations:** 1 Department of Neurology and Clinical Neuroscience, Yamaguchi University Graduate School of Medicine, Ube 7558505, Yamaguchi, Japan; kogamrk@yamaguchi-u.ac.jp (M.K.); ynanami@yamaguchi-u.ac.jp (N.Y.); mnakamor@yamaguchi-u.ac.jp (M.N.); 2 Faculty of Medicine and Health Sciences, Yamaguchi University Graduate School of Medicine, Ube 7558505, Yamaguchi, Japan

**Keywords:** Guillain–Barré syndrome, small nuclear ribonucleoprotein autoantibodies, blood–nerve barrier, MFS/GBS overlap syndrome

## Abstract

We recently identified the U1 small nuclear ribonucleoprotein (U1-snRNP) antibodies in patients with Guillain–Barré syndrome (GBS), which is associated with the breakdown of the blood–nerve barrier (BNB). The objective of this study was to clarify the clinical significance of U1-snRNP antibodies in patients with GBS and its variants. We measured U1-snRNP antibodies using an enzyme-linked immunosorbent assay from the serum samples of patients with GBS (n = 106), Miller Fisher syndrome (MFS) (n = 24), and MFS/GBS overlap syndrome (MFS/GBS, n = 8). We compared the clinical characteristics of U1-snRNP positive and U1-snRNP negative GBS patients (n = 106). The cerebrospinal fluid (CSF)/serum albumin quotient (QALB)/QALBLIM [calculated as(age/15) + 4)] was calculated. The prevalence of U1-snRNP antibody positivity was 39% (41/106) in GBS, 0% (0/24) in MFS, and 50% (4/8) in MFS/GBS. The rate of U1-snRNP antibody positivity in the GBS and MFS/GBS groups was significantly higher than that in the MFS group. Levels of CSF proteins and QALB/QALBLIM were higher in U1-snRNP antibody-positive GBS than in U1-snRNP antibody-negative GBS among all GBS patients, as well as GBS patients with a preceding *Campylobacter jejuni* infection or AIDP. In conclusion, the U1-snRNP antibody-positive GBS group had a more severe breakdown of the BNB in U1-snRNP antibody-positive GBS patients than in U1-snRNP-negative GBS patients. The presence of U1-snRNP antibodies may be a clinical biomarker for predicting the progression of MFS to MFS/GBS.

## 1. Introduction

Guillain–Barré syndrome (GBS) is an acute immune-mediated neuropathy characterized by rapidly progressive limb weakness after antecedent infection [1]. The pathogenesis of GBS is explained by molecular mimicry between microbial antigens and gangliosides at the peripheral nerve membrane [2]. GBS is characterized by increased protein levels in the cerebrospinal fluid (CSF), the presence of demyelination and/or axonal damage in a nerve conduction study (NCS), and positivity for anti-ganglioside antibodies [3,4]. *Campylobacter jejuni*, *Haemophilus influenzae*, severe acute respiratory syndrome coronavirus 2 (SARS-CoV-2) virus, Epstein–Barr virus (EBV), cytomegalovirus (CMV), *Mycoplasma pneumoniae*, influenza A virus, hepatitis E virus, and Zika virus have been reported as the most common causes of antecedent infections in GBS [5].

Miller Fisher syndrome (MFS) is a subtype of GBS characterized by three main symptoms: extraocular muscle palsy, diminished tendon reflexes, and ataxia [6]. It typically develops following a preceding infection and follows a monophasic course, with symptoms resolving spontaneously after 1–2 weeks [6]. GQ1b antibodies, which are glycolipid antibodies, are detected in many cases [7]. MFS/GBS overlap syndrome (MFS/GBS) is a condition in which the clinical features of MFS are combined with those of GBS, such as muscle weakness in the limbs [8,9]. Bickerstaff brainstem encephalitis (BBE) is a central nervous system disorder characterized primarily by impaired consciousness, extraocular muscle palsy, and ataxia; it has similar symptoms to MFS and shares a common immunological pathogenesis mediated by serum anti-GQ1b antibodies [10]. Rare cases of BBE overlapping with GBS (BBE/GBS) have been reported, supporting the view that these conditions are part of a continuous clinical spectrum [11].

Previous observations have reported disruption of the blood–nerve barrier (BNB) in patients with Chronic Inflammatory Demyelinating Polyradiculoneuropathy (CIDP) and GBS [12,13]. We recently reported that U1 small nuclear ribonucleoprotein (U1-snRNP) autoantibodies are associated with the breakdown of the BNB in GBS. IgG from U1-snRNP antibody-positive patients significantly reduced barrier function and claudin-5 expression in an in vitro BNB co-culture model compared to IgG from healthy controls (HCs) [13]. 

Clinical parameters reflecting BNB disruption include elevated cerebrospinal fluid protein levels and QALB [14,15,16,17]. Elevated CSF protein levels reflect inflammation of the central nervous system, such as meningitis, encephalitis, and Guillain–Barré syndrome, increased permeability of the blood–CSF barrier, and disorders of CSF circulation and absorption [17]. In addition, elevated QALB/QALBLIM levels reflect age-adjusted damage to the blood–CSF barrier [14,15,16]. A QALB/QALBLIM ratio of ≤1 indicates no BNB disruption; <2 indicates mild BNB disruption; and ≥2 indicates severe BNB disruption [14].

In the present study, we evaluated the association between U1-snRNP antibody positivity and clinical information in 106 GBS patients (41 patients with U1-snRNP antibodies and 65 patients without U1-snRNP antibodies). We also assayed U1-snRNP autoantibodies in serum samples from MFS and MFS/GBS.

## 2. Results

### 2.1. Differences in Clinical Profiles Between U1-snRNP Antibody-Positive and -Negative GBS

The rate of U1-snRNP antibody positivity was 39% (41/106) in GBS patients and 0% (0/24) in MFS. The cutoff value for determining U1-snRNP antibody positivity and negativity was defined based on previous data [13]. Since previous studies have reported that the prevalence of U1-snRNP antibodies in healthy individuals is 0% (0/16), and that the positivity rate in disease controls (DCs) is also low at 0.017% (2/113), U1-snRNP antibodies were not measured in healthy individuals in the present study [13].

Elevated QALB/QALBLIM levels reflect age-adjusted disruption of the blood–CSF barrier, while cerebrospinal fluid protein levels reflect increased permeability of the blood–CSF barrier and inflammation within the subarachnoid space. Levels of both proteins in the CSF and QALB/QALBLIM were higher in U1-snRNP antibody-positive GBS than in U1-snRNP antibody-negative GBS (Table 1). There were no marked differences between the groups in age, sex, peak GDS, ΔGDS and the rate of GDS ≥ 5, antecedent infection, cells in CSF, IgG index, electrophysiological diagnosis (e.g., AIDP or AMAN), or rate of anti-ganglioside antibody positivity (Table 1).

### 2.2. The Rates of U1-snRNP Antibody Positivity Among the Clinical Phenotypes of GBS

The rates of U1-snRNP antibody positivity according to the clinical phenotypes of GBS were as follows: GBS patients, 39% (37/96); MFS/GBS, 50% (4/8); MFS, 0% (0/24); and GBS/BBE, 0% (0/2) (Table 2). The rate of U1-snRNP antibody positivity in the GBS group was significantly higher than that in the MFS group (Table 2). Importantly, the rate of U1-snRNP antibody positivity in the MFS/GBS group was significantly higher than that in the MFS group (Table 2).

### 2.3. Differences in Clinical Profiles Between U1-snRNP Antibody-Positive and -Negative C. jejuni GBS or AIDP

Among GBS patients with a preceding *C. jejuni* infection, there were no significant differences in peak GDS between U1-snRNP antibody-positive GBS and U1-snRNP antibody-negative GBS; however, protein levels in the CSF and QALB/QALBLIM were both significantly higher in U1-snRNP antibody-positive GBS than in U1-snRNP antibody-negative GBS (Table 3). Among AIDP patients defined using both Rajabally’s criteria and anti-ganglioside antibody negativity, proteins levels in the CSF and QALB/QALBLIM were both significantly higher in U1-snRNP antibody-positive GBS than in U1-snRNP antibody-negative GBS (Table 3). In contrast, peak GDS was significantly lower in U1-snRNP antibody-positive GBS than in U1-snRNP antibody-negative GBS (Table 3).

## 3. Discussion

U1-snRNPs play a major role in pre-mRNA splicing by removing introns from messenger RNA precursors, forming mature mRNA [18]. The dysregulation of U1-snRNP components has been associated with several disorders, including neurodegeneration (spinal muscular atrophy [SMA], fused in sarcoma [FUS]-mediated amyotrophic lateral sclerosis [ALS], frontotemporal dementia, and Alzheimer’s disease [AD]), cancer, and autoimmune disease [19]. The pathogenic cause of SMA is a reduction in survival motor neuron (SMN) proteins due to the mutation of the *SMN1* gene, resulting in motor axon degeneration caused by a reduction in snRNP biogenesis [20]. Aberrant interaction of FUS-mediated ALS with U1 snRNA also leads to impaired snRNP biogenesis, suggesting a molecular link between SMA and FUS-ALS [21]. In AD, U1-snRNP splicing dysfunction causes AD pathogenesis, including neuronal hyperexcitability and cognitive impairment [22]. Furthermore, previous in vitro research showed that a decrease in U1-snRNP activates and accelerates the migration and invasion of cancer cells [23].

**Figure 1:**
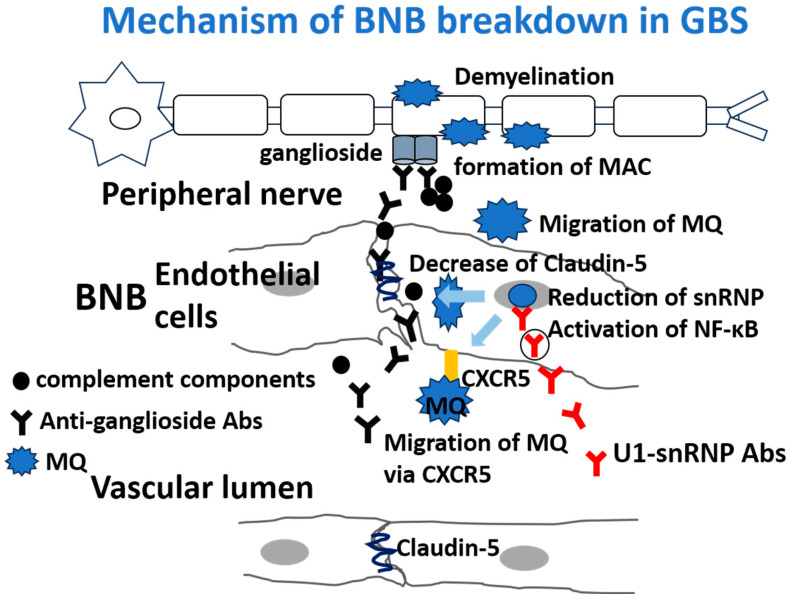
Mechanism of BNB breakdown in GBS. U1-snRNP antibodies (U1-snRNP Abs) cause BNB breakdown via a reduction in U1-snRNP and induction of NF-κB activation, leading to decrease in claudin-5 and increase in C-X-C Motif Chemokine Receptor 5 (CXCR5). Increased BNB permeability leads to the entry of anti-ganglioside Abs and complement components into peripheral nerves, resulting in anti-ganglioside Ab- and complement-mediated injury [formation of the membrane attack complex (MAC)] in peripheral nerves. Migration of macrophages (MQ) via CXCR5 causes peripheral nerve demyelination.

U1-snRNP autoantibodies have been detected in the sera of patients with several collagen diseases, including mixed connective tissue disease (MCTD), systemic sclerosis, systemic lupus erythematosus (SLE), and myositis. The presence of U1-snRNP antibodies is clinically useful for the diagnosis of MCTD [24,25,26], although it remains unclear whether these antibodies are pathogenic. Previous studies showed that U1-snRNP antibodies can bind to U1 RNP on the cell surface and initiate inflammation in endothelial cells from the pulmonary artery via the upregulation of ICAM-1 and E-selectin [27,28]. Our previous study suggested that U1-snRNP antibodies in GBS-IgG lead to a decrease in U1-snRNP and induce the activation of NF-κB in human BNB-endothelial cells as the upstream regulator, resulting in increased permeability of the BNB in GBS (Figure 1) [13].

Previous research suggests that the production of U1-snRNP autoantibodies can be triggered by several viral infections, including CMV, HHV-6, HHV-7, HIV, and SARS-CoV-2, through molecular mimicry [29,30,31,32]. In particular, the molecular homology between the epitopes of CMV glycoprotein B and the charged amino acids in the carboxy terminal region of the U1-70K component of U1-snRNP has been proposed as a mechanism underlying the production of U1-snRNP antibodies in response to U1-snRNP [29]. HHV-6, HHV-7, and EBV also possess proteins of glycoprotein B homologous to that of CMV [29]. Our previous study demonstrated that the positivity rate of U1-snRNP antibodies was 36% (28 of 77) in GBS patients, 7% (2 of 28) in DCs, and 0% (0 of 16) in HCs [13]. In addition, antecedent infection with *Campylobacter jejuni*, CMV, or EBV is particularly associated with GBS, and antecedent infections have been identified in U1-snRNP antibody-positive GBS cases [13]. We speculated that the periodic response to U1-snRNP from antecedent infection with pathogens in GBS accelerated the temporary production of U1-snRNP antibodies [13].

In the present study, U1-snRNP antibody-positive GBS showed significantly higher protein levels in the CSF and increased QALB/QALBLIM relative to U1-snRNP antibody-negative GBS, reflecting a more severe breakdown of the BNB in patients with U1-snRNP antibody-positive GBS than in patients with U1-snRNP antibody-negative GBS, although no significant difference in disease severity was observed between the two groups. In addition, no significant association with antecedent infection was observed between the U1-snRNP antibody-positive and -negative groups. All MFS patients were negative for U1-snRNP antibodies, but 39% of GBS patients and 50% of MFS/GBS patients were positive for U1-snRNP antibodies, possibly suggesting a surrogate marker that predicts the progression of MFS/GBS from MFS.

In the present study, protein levels in the CSF and QALB/QALBLIM were both increased in U1-snRNP antibody-positive GBS in comparison to U1-snRNP antibody-negative GBS among GBS patients with preceding *C. jejuni* infection, suggesting that U1-snRNP antibodies are associated with the breakdown of the BNB in *C. jejuni* infection-related axonal GBS. To date, there are no reports on whether U1-snRNP antibodies are produced in GBS patients with preceding *C. jejuni* infection due to molecular mimicry, but the present study is the first to suggest an association between *C. jejuni* infection and the production of U1-snRNP antibodies. In addition, in the present study, protein levels in both the CSF and QALB/QALBLIM were increased in U1-snRNP antibody-positive GBS in comparison to U1-snRNP antibody-negative GBS among AIDP patients, suggesting that U1-snRNP antibodies are associated with the breakdown of the BNB in not only axonal GBS but also AIDP. Based on the results of the present study, we assumed that a temporary autoantibody response against U1-snRNP could be boosted by the periodic response to cell destruction induced by *C. jejuni* infection or viral infection in GBS, and that such individuals may be at risk of developing GBS within a relatively short period of time. Based on the result of the present study that the peak GDS was lower in U1-snRNP antibody-positive AIDP than in U1-snRNP antibody-negative AIDP, we speculated that there may be factors other than BNB breakdown that determine the severity of GBS in AIDP patients.

The main limitation of this study is the absence of multicenter, blind investigations; therefore, further research is warranted. Another limitation is that, although no significant correlation was found between the degree of BNB damage and GBS severity as defined by the GDS, since BNB failure is thought to be associated with more severe GBS, the study did not examine other prognostic factors beyond the GDS (e.g., whether the patient was able to walk at 6 months). Furthermore, although it has been proposed that U1-snRNP antibodies may serve as a biomarker for predicting the progression of MFS to the more severe MFS/GBS overlap syndrome, MFS/GBS overlap syndrome is a rare disease with a small number of cases; therefore, future studies with a larger sample size are necessary.

In conclusion, the present study shows that U1-snRNP antibody-positive GBS patients had more severe BNB breakdown than U1-snRNP antibody-negative GBS patients. U1-snRNP antibody positivity was observed more frequently in patients with GBS or MFS/GBS than in patients with MFS, suggesting a possible surrogate marker that predicts the progression of MFS/GBS from MFS. An association between U1-snRNP antibody positivity and BNB breakdown was observed in GBS patients with preceding *C. jejuni* infection and AIDP.

## 4. Methods

### 4.1. Study Protocol Approval, Registrations, and Patient Consent

This study was approved by the Ethics Committee of the Yamaguchi University School of Medicine (IRB No.: 2024-016; Approval Date: 24 April 2024). Written informed consent was obtained from all the participants.

### 4.2. Serum Samples

We collected sera from 106 GBS patients, 24 MFS patients, and 8 MFS/GBS patients, overlapping Guillain–Barré syndrome and Bickerstaff brainstem encephalitis (GBS/BBE) patients during the acute phase—after the onset of muscle weakness and before the start of treatment. All of these patients were examined at Yamaguchi University Hospital or referred to this hospital to assess the presence of anti-glycolipid antibodies in their serum. Serum samples were stored at −80 °C until use.

### 4.3. Clinical Information of GBS Patients

We collected the following information from GBS patients: sex, age, GBS Disability Scale (GDS) on admission/peak, ΔGDS scale (peak GDS−admission GDS), causative antecedent infection (*C. jejuni*, *H. influenzae*, CMV, EBV, *M. pneumoniae,* or SARS-CoV-2) [33], serum anti-ganglioside antibodies (anti-GM1, GalNAc-GD1a, GD1a, GQ1b, or GT1a IgG antibodies) [34], CSF data (cell count, total proteins, IgG index, and QALB/QALBLIM), and electrophysiological diagnosis of acute inflammatory demyelinating polyradiculoneuropathy (AIDP) and acute motor axonal neuropathy (AMAN) from a nerve conduction study. Both QALB (CSF/serum albumin ratio) and QALBLIM [(age/15) + 4] were calculated for functional assessment of blood–spinal nerve root barrier (B-SNR-B) permeability [14]. The diagnostic criteria for the initial NCS were based on Rajabally’s criteria [35].

### 4.4. Detection of U1-snRNP Antibodies in GBS Patients Using ELISA

Sera from 106 GBS patients and 24 MFS patients were used for enzyme-linked immunosorbent assay (ELISA; Anti-RNP-70, RUO632, Sebia, Lisses, France). Serum samples were tested according to the manufacturer’s instructions, as previously described [13].

Diluted serum samples were added to a 96-well plate coated with RNP-70. After incubation for 30 min on a shaker at room temperature (RT), the liquid in the wells was discarded, and the wells were washed three times. Next, an enzyme conjugate containing horseradish peroxidase (HRP)-labeled anti-human IgG antibody was added, and the plate was incubated for 15 min. After incubation with tetramethylbenzidine substrate solution for 15 min, a stop solution was added. The absorbance of the wells at 450 nm was measured using an ELISA plate reader (Molecular Devices, CA, USA). A calibration curve was constructed by plotting the concentration of the calibrator on the *x*-axis (logarithmic scale) and the absorbance of the calibrator on the *y*-axis (linear scale).

### 4.5. Statistical Analyses

Prism 7 (GraphPad) was used for all statistical analyses. For pairwise comparisons, we used the unpaired Student’s *t*-test (two-tailed); for comparisons of categorical variables, we used the two-sample test for differences in population proportions. A *p*-value of 0.05 or less was considered statistically significant.

## Figures and Tables

**Table 1:** Differences in clinical profiles between U1-snRNP antibody-positive and -negative GBS.

GBS	n	Age (SD)	Sex (M/F)	GDS		
				GDS in peak	ΔGDS	GDS 5
U1-snRNP (+)	41	56 (±20)	21/20	4 (±1)	4 (±1)	29% (8/41)
U1-snRNP (−)	65	54 (±22)	37/28	4 (±1)	3 (±1)	20% (13/65)
*p* value		* p = 0.596	^#^ *p* = 0.689	* *p* = 0.447	* *p* = 0.765	^#^ *p* > 0.999
GBS	Antecedent infection					
	*C. Jejuni*	*H. Influenzae*	CMV	EBV	*M. pneumoniae*	SARS- CoV-2
U1-snRNP (+)	24% (10/41)	7% (3/41)	2% (1/41)	2% (1/41)	2% (1/41)	2% (1/41)
U1-snRNP (−)	38% (25/65)	3% (2/65)	9% (6/65)	0% (0/65)	6% (4/65)	0% (0/65)
*p* value	^#^ *p* = 0.145	^#^ *p* = 0.373	^#^ *p* = 0.244	^#^ *p* = 0.387	^#^ *p* = 0.647	^#^ *p* = 0.389
GBS	CSF					
	CSF cells (/μL)	CSF Protein (mg/dL)	IgG index		QALB/ QALBLIM	
U1-snRNP (+)	4.62 (±8.63)	100.8 (±89.6)	0.55 (±0.15)		2.16 (±1.77)	
U1-snRNP (−)	2.32 (±2.17)	58.4 (± 33.7)	0.52 (±0.12)		0.85 (±0.66)	
*p* value	* *p* = 0.067	* *p* = 0.002	* *p* = 0.516		* *p* = 0.017	
GBS	NCS		Anti-ganglioside antibodies			
	AMAN	AIDP	GM1, GD1a, GalNAc-GD1a		GT1a, GQ1b	negative
U1-snRNP (+)	61% (19/31)	39% (12/31)	43% (17/40)		13% (5/40)	45% (18/40)
U1-snRNP (−)	65% (31/47)	34% (16/47)	44% (28/64)		19% (12/64)	37% (24/64)
*p* value	^#^ *p* = 0.89		^#^ *p* = 0.667 (GM1, GD1a, GalNAc-GD1a vs. negative)			
			^#^*p* = 0.391 (GT1a, GQ1b vs. negative)			

Data are expressed as mean (±SD) or % (number). GBS, Guillain–Barré syndrome; M, male; F, female; GDS, GBS Disability Scale, ΔGDS; peak GDS−admission GDS; *C. jejuni*, *Campylobacter jejuni*; *H. influenzae*, *Haemophilus influenzae*; *M. pneumoniae*, *Mycoplasma pneumoniae*; SARS-CoV-2, severe acute respiratory syndrome coronavirus 2; U1-snRNP(+), U1-snRNP antibody-positive GBS; U1-snRNP(−), U1-snRNP antibody-negative GBS; CSF, cerebrospinal fluid; IgG, immunoglobulin G; AMAN, acute motor axonal neuropathy; AIDP, acute inflammatory demyelinating polyradiculoneuropathy; GM1, GD1a, and GalNAc-GD1a, positive for anti-ganglioside antibodies against GM1, GD1a, or GalNAc-GD1a; GT1a, GQ1b, positive for anti-ganglioside antibodies. Data were analyzed using an unpaired Student’s *t*-test (two-sided) for pairwise comparisons (*p* = *) or by testing two samples for differences in population proportions to compare categorical variables (*p* = #). Bold text indicates a statistically significant difference.

**Table 2:** The rates of U1-snRNP antibody positivity among the clinical phenotypes of GBS.

	Clinical phenotype			
	GBS	GBS/MFS	MFS	GBS/BBE
U1-snRNP (+)	39% (37/96)	50% (4/8)	0% (0/24)	0% (0/2)
*p* value	***p* = 0.0019 (GBS/MFS vs. MFS)**			
	*p* = 0.708 (GBS vs. GBS/MFS)			
	***p*** **< 0.0001 (GBS vs. MFS)**			

Data are expressed as % (number). GBS, Guillain–Barré syndrome; GBS/MFS, Miller Fisher/Guillain–Barré overlap syndrome; MFS, Miller Fisher syndrome; GBS/BBE, overlapping Guillain–Barré syndrome and Bickerstaff brainstem encephalitis; U1-snRNP(+), U1-snRNP antibody-positive GBS. Data were analyzed by testing two samples for differences in population proportions to compare categorical variables. Bold text indicates a statistically significant difference.

**Table 3:** Differences in clinical profiles between U1-snRNP antibody-positive and -negative *C. jejuni* GBS or AIDP.

*C. jejuni* GBS	n	Peak GDS	CSF Protein (mg/dL)	QALB/ QALBLIM
U1-snRNP (+)	6	3 (±1)	111.3 (±86.9)	3.34 (±1.74)
U1-snRNP (−)	19	4 (±1)	50.4 (±25.4)	0.57 (±0.22)
*p* value		*p* = 0.5192	***p*** **= 0.0161**	***p*** **= 0.0225**
AIDP	n	peak GDS	CSF protein (mg/dL)	QALB/ QALBLIM
U1-snRNP (+)	10	3 (±1)	157.5 (±105.3)	2.85 (±1.65)
U1-snRNP (−)	10	4 (±1)	61.3 (±33.4)	1.23 (±0.85)
*p* value		***p*** **= 0.0232**	***p*** **= 0.0177**	***p*** **= 0.0232**

Data are expressed as mean (±SD) or % (number). *C. jejuni*, *Campylobacter jejuni*; GBS, Guillain–Barré syndrome; GDS, GBS Disability Scale; CSF, cerebrospinal fluid; AIDP, acute inflammatory demyelinating polyradiculoneuropathy. Data were analyzed using an unpaired Student’s *t*-test (two-sided) for pairwise comparisons. Bold text indicates a statistically significant difference.

## Data Availability

The original contributions presented in this study are included in the article. Further inquiries can be directed to the corresponding author(s).
